# Correlations between drug use, HIV disclosure and interpersonal communication on sexual risk behaviour of HIV-positive men who have sex with men

**DOI:** 10.1186/s12919-020-00200-5

**Published:** 2020-12-08

**Authors:** Lussy Afriyanti, Agung Waluyo, Sri Yona

**Affiliations:** 1 Master of Nursing, Speciality in Medical Surgical Nursing, Faculty of Nursing, Universitas Indonesia, Depok, Indonesia; 2 Technical Training Section, Balai Pelatihan Kesehatan Batam, Batam, Indonesia; 3 Medical Surgical Nursing Department, Faculty of Nursing, Universitas Indonesia, Depok, Indonesia

**Keywords:** Drug use, HIV disclosure, Interpersonal communication, Men who have sex with men, Sexual risk behaviour

## Abstract

**Background:**

The Human Immunodeficiency Virus (HIV) epidemic is a global health problem whose number of cases are always higher among men who have sex with men (MSM). Most existing MSM have moderate and very high risk behaviour in HIV transmission. This study was designed to identify correlations between drug use, HIV disclosure and interpersonal communication patterns on sexual risk behaviour among HIV-positive MSM.

**Methods:**

This study used a cross sectional design with a purposive sampling technique for participants who visited the voluntary counselling and testing (VCT) clinic in a referral hospital and snowball sampling technique for participants in the work area of a community health centre in Batam involving 126 HIV-positive MSM. Data were collected by 5 part questionnaire, namely demographic questionnaire, drug screening questionnaire, brief scale for HIV self disclosure, communication pattern questionnaire-short form, and safe sex behaviour questionnaire. Bivariate analysis was applied to determine whether there is a relationship between drug use, HIV disclosure, interpersonal communication and demographic characteristics (ethnicity, educational status) with sexual risk behaviour of MSM. Logistic regression analysis was used to explore the variables that most associated variable to sexual risk behaviour.

**The results:**

The results showed that there was significant correlation between HIV disclosure and sexual risk behaviour (*p* = 0.019, α = 0.05, OR = 2.530) and significant correlation between interpersonal communication patterns and sexual risk behaviour (*p* = 0.016, α = 0.05, OR = 2.589). There is no significant correlation between demographic characteristics, namely: ethnicity and educational status with sexual behaviour at risk of MSM. In multiple logistic regression analysis, educational status was the factor that most associated with sexual risk behaviour among HIV-positive MSM (*p* = 0.027, α = 0.05, OR = 2.807, 95% CI = 1.125–7.006).

**Conclusions:**

HIV disclosure and interpersonal communication patterns have a significant negative correlation with sexual risk behaviour among HIV-positive MSM. MSM with low HIV disclosure have high risk sexual behaviour opportunities. MSM with the closed interpersonal communication pattern also has a high risk of sexual behaviour. Education status was the most associated variable to sexual risk behaviour of HIV-positive MSM. Nurses as professional health workers need to improve comprehensive assessment, personal counselling and plan specific learning model by involving HIV-positive MSM in reducing HIV transmission from risky behaviour.

## Background

The HIV epidemic is a rapidly developing global health problem until now. UNAIDS data in 2017 showed that 36.9 million people in the world lived with HIV/AIDS. In Indonesia, 620,000 people lived with HIV/AIDS, and 38,000 people died from AIDS in 2016 [1]. In Batam, Kepulauan Riau Province, there were 6141 people cumulatively recorded as being infected with HIV between 1992 to June 2018. In the last 6 years, HIV testing of high-risk groups has shown increased cases of new infections of more than 500 people each year [2]. In 2017, among 540 men who have sex with men (MSM) and who visited VCT clinics in Batam, there were 221 new HIV-positive MSM [3]. The risk of HIV infection was 27 times higher among MSM [4], the prevalence of HIV cases in MSM ranges from 5 to 25% and showed a consistent increasing trend from 2011 to 2015 [5].

Based on the behavioural analysis examined by Rocha, et al. [6], most MSM was found to have moderate and very high risk behaviour for HIV infection [6]. Among the factors that can influence sexual behaviour at risk of MSM in HIV transmission are drug use, disclosure of HIV status and interpersonal communication patterns of MSM with their sexual partners. The prevalence of drug use among HIV-positive MSM tends to be higher, and they even tend to use more than one substance (polydrug). A study in Austrian found that the prevalence of drug use among HIV-positive MSM was 60.5% and for polydrug use, it was 69.4%, and approximately 42.4% of drug users had unprotected sex [7]. Polydrug use of HIV diagnosed MSM is associated with an increased prevalence of unprotected anal intercourse (UAI), both with seroconcordant or serodiscordant couples, and a higher risk of HIV infection [8].

Another factor associated with the risk of HIV transmission and exposure is HIV disclosure. Disclosure of HIV-positive MSM was still low [9, 10]. An online cross-sectional survey conducted on HIV-positive MSM in Asia found that only 7% revealed their HIV status to all their partners [9]. Based on a study in Bangkok, the level of disclosure was low at least only 26% of sexual partners in the last sexual relationship [10]. The probability of exposure to HIV through anal intercourse (AI) without condom use is known to be substantially lower after serostatus disclosure compared to nondisclosure [11]. However, in HIV-positive MSM with low disclosure, UAI was low (17%), where half were with permanent partners [10]. In contrast, research in the United States revealed that HIV-positive MSM were more likely to engage in UAI when disclosing their status to their sexual partners, because disclosure was used as an attempt to allow unprotected sexual intercourse with partners who were also HIV positive [12].

Nonverbal communication and signals expressing sexual orientation that allows the MSM to recognize each other is dominant among this community [13]. A cross-sectional survey of 3588 MSM in China found that most communication between MSM (79.1%) occurred via the internet [14]. The technology of social-sexual networking has been well integrated into the lives of MSM and facilitates anonymous sexual meetings and meetings with new partners [15]. Communication with MSM partners, especially before sexual intercourse can result in safer sexual practices that reduce the risk of HIV transmission [16].

Limited research has been conducted in this area in Indonesia, especially in Batam. One of the qualitative studies conducted in Makassar aimed to identify the effect of drug abuse among gay men on sexual risk behaviour; results suggested that the use of drugs increased the tendency for condomless intercourse, sexual intercourse with nonpermanent partners, and AI [17]. Research on HIV disclosure has been conducted in Medan, and the results demonstrated no significant relationship between HIV disclosure and sexual risk behaviour in HIV-positive MSM [18]. The relationship between drug use, HIV disclosure, interpersonal communication patterns and sexual risk behaviour in HIV-positive MSM is important to examine in order to determine the influence of local wisdom in drug use, HIV disclosure, interpersonal communication patterns, and sexual risk behaviour of MSM.

## Methods

### Study design and participants

This research used a cross-sectional design to determine the correlation between drug use, HIV disclosure, and interpersonal communication patterns towards sexual risk behaviour. Samples included HIV-positive MSM who visited the VCT clinic; 56 purposive sampling of participants who visited the VCT clinic in a referral hospital in Batam and 70 snowball sampling of participants in the work area of a community health centre in Batam were used to obtain adequate participants for this study. This was because of the difficulty in obtaining samples from people who visited the community health centre. The inclusion criteria for the sample were HIV-positive MSM (based on the initial screening questionnaire); aged ≥18 years; able to read and write in Bahasa; and did not experience mental disorders determined based on the patient’s medical record. While the exclusion criteria for the sample in this study were HIV-positive MSM who were current during the stay in the hospital or clinic because it will affect the results of the measurement of the research variables to be studied.

### Data collection

Data collection was conducted using questionnaires with the assistance of enumerators who were the counsellors and peer of people living with HIV/AIDS (PLWHA). The data collection tool consisted of five parts; namely, demographic questionnaire, the drug screening questionnaire (DAST), brief scale for HIV self disclosure, communication pattern questionnaire-short form (CPQ-SF), and safe sex behaviour questionnaire (SSBQ) [19–22]. Validity and reliability tests were carried out on the DAST, CPQ-SF and SSBQ questionnaires against 30 respondents living with HIV / AIDS in one of the hospitals in Batam. The results of the validity test of each questionnaire obtained the value of r table = 0.3061 for α = 0.05 and degrees of freedom n-2 and the number of samples *n* = 30. The reliability test results of the DAST questionnaire obtained Cronbach’s Alpha value of 0.821 (Cronbach’s Alpha ≥0, 6). The CPQ-SF reliability test obtained a Cronbach’s Alpha value of 0.802 (Cronbach’s Alpha ≥0.6), while the SSBQ reliability test obtained a Cronbach’s Alpha value of 0.850 (Cronbach’s Alpha ≥0.6). The Brief Scale for HIV Self Disclosure used in this study was tested for validity with a Cronbach’s Alpha value of 0.73.

Researchers approached participants through three enumerators. Two enumerators at the hospital provided information about research to HIV-positive MSM patients who visited the VCT clinic and requested the patient’s permission to participate. One other enumerator visited HIV-positive MSM patients who were in the work area of a community health centre and provided information about the research and asked MSM permission to participate. Each participant who was willing to participate in this study was given a more complete explanation by the enumerator, and the participant was asked to sign an informed consent form. In this study, some patients were unwilling to participate on the grounds that they were only permitted a brief period to take medicine and had to return to work immediately. Two participants had to stop during the survey because not willing to report their drug use status and sexual behaviour. Participants who had completed the questionnaires received a reimbursement of transportation money to their homes of IDR 50,000. Ethical approval from The Ethics Committee Faculty of Nursing Universitas Indonesia by number 05./UN2.F12.D/HKP.02.04/2019 was obtained for this study before data collection started.

### Sexual risk behaviour

Sexual risk behaviour was sexual activity that can increase the risk of contracting HIV infection. Sexual risk behaviour was measured using an SBBQ questionnaire consisting positive and negative statement items. High-risk sexual behaviour was if the total score is less than the cutoff point using the mean value.

Demographic characteristics were collected, including age, ethnicity, employment, monthly income, educational status, marital status and length of HIV diagnose.

### Data analysis

The data were cleaned and entered into a computer for analysis using the SPSS analysis program version 23. Univariate analyses were performed using descriptive statistics on the demographic characteristics, drug use, HIV disclosure, interpersonal communication and sexual risk behaviour. Bivariate analysis by the Chi Square non-parametric test to determine correlation between independent and dependent variable, significance was concerned at *p*-value < 0.05 with 95% confidence interval. The independent variables in this study were drug use, HIV disclosure and interpersonal communication, while the independent variables of demographic characteristics were ethnicity and educational status. The sexual risk behaviour was the dependent variable of this study. Multivariate analysis by using logistic regression analysis to determine correlation of several independent variables with a category dichotomous dependent variable.

## Results

### Characteristics of participants

A total 128 HIV-positive MSM were invited to participate in this study. There were two participants not willing to report their drug use status and sexual behaviour, 126 HIV-positive MSM was involved in the final analysis. The results of the study found the mean age of participants was 30.63 years (SD = 7.247), the mean length of time diagnosed with HIV was 24.25 months (SD = 27.075). Of 126 participants, almost all (90.5%) were young adults, more than three-quarters (77.0%) of MSM were non-Malay or immigrant in Batam. Almost all of the participants (95.2%) were employed and had high income (83.3%). Most (77.8%) participants had low education levels, almost of MSM were unmarried or widowers (92.1%) and more than three-fifths (67.5%) had been diagnosed with HIV for ≥12 months. More than three-fifth (61.9%) of MSM were at no risk/low risk of using drugs. More than half (56.3%) had low disclosure, 52.4% had an open interpersonal communication pattern. More than half of participants 56.3% had high sexual risk behaviour (Table 1).

**Table 1:** Characteristics of HIV-positive MSM (*n* = 126)

Characteristics	Number	Percent
*Age*		
a. Young Adults (18–39 years old)	114	90.5%
b. Middle Adults (40–65 years old)	12	9.5%
*Ethnicity*		
a. Malay	29	23.0%
b. Non Malay	97	77.0%
*Employment*		
a. Employed	120	95.2%
b. Unemployed	6	4.8%
*Monthly income*		
a. Low	21	16.7%
b. High	105	83.3%
*Educational status*		
a. Low (Senior high school and below)	98	77.8%
b. High (College and above)	28	22.2%
*Marital status*		
a. Single/widower	116	92,1%
b. Married	10	7.9%
*Length of diagnose*		
a. < 12 months	41	32.5%
b. ≥ 12 months	85	67.5%
*Drug use*		
a. Risk	48	38.1%
b. No risk/ low	78	61.9%
*HIV disclosure*		
a. Low	71	56.3%
b. High	55	43.7%
*Interpersonal communication*		
a. Closed pattern	60	47.6%
b. Open pattern	66	52.4%
Sexual risk behaviour		
a. High risk	71	56.3%
b. Low risk	55	43.7%

High monthly income: if the income is higher and equal than the Riau Islands Province minimum wage 2018 (income ≥ IDR 2,563,875)

High HIV disclosure: if the total score measured by the Brief Scale for HIV Self Disclosure ≥7

### Bivariate analysis

Chi square test demonstrated no significant correlation between drug use (*p* = 1.000, α = 0.05, OR = 0.994) with sexual risk behaviour. Variables of HIV disclosure (*p* = 0.019, α = 0.05, OR = 2.530) and interpersonal communication patterns (*p* = 0.016, α = 0.05, OR = 2.589) exhibited a significant negative correlation with sexual risk behaviour (Table 2). Participants with low HIV disclosure had a 2.530 times greater probability of experiencing high risk sexual behaviour compared with participants with high HIV disclosure. Participants who had closed communication patterns had 2589 times the opportunity of experiencing high-risk sexual behaviour compared to participants who had an open communication pattern. Statistical test results showed no significant correlation between ethnicity and educational status with sexual risk behaviour (Table 3).

**Table 2:** Analysis the correlation of drug use, HIV disclosure and interpersonal communication patterns to sexual risk behaviour (*n* = 126)

Variable	Sexual risk behaviour						OR (95%CI)	*P value*
	Low risk		High risk		Total			
	N	%	N	%	N	%		
Drug use								
No risk/Low	34	43.6	44	56.4	78	100	0.994 (0.481–2.051)	1.000
Risk	21	43.8	27	56.3	48	100		
HIV disclosure								
High disclosure	31	56.8	24	43.6	55	100	2.530 (1.225–5.223)	0.019*
Low disclosure	24	33.8	47	66.2	71	100		
Interpersonal communication								
Open pattern	36	54.5	30	45.5	66	100	2.589 (1.250–5.365)	0.016*
Closed pattern	19	31.7	41	68.3	60	100		

*OR* odds ratio, *CI* confidence interval, *significant at α = 0.05

**Table 3:** Analysis the correlation of demography characteristic to sexual risk behavior (*n* = 126)

Variable	Sexual risk behavior						OR (95%CI)	*P value*
	Low risk		High risk		Total			
	N	%	N	%	N	%		
Ethnicity								
Malay	45	53,6	52	53,6	97	100	1.644 (0.693–3.899)	0.357
Non-Malay	10	34,5	19	65,5	29	100		
Educational status								
High	17	60.7	11	39.3	28	100	2.440 (1.032–5.769)	0.065
Low	38	38.8	60	61.2	98	100		

*OR* odds ratio, *CI* confidence interval

**Table 4:** Final modelling result

Variabel	*P value*	OR	95%CI
Second model			
HIV disclosure	0.014	2.609	1.215–5.606
Interpersonal communication	0.017	2.527	1.179–5.418
Education status	0.027	2.807^a^	1.125–7.006
Constanta	0.001	0.241	

*OR* odds ratio, *CI* confidence interval

^a^greatest OR value

### Multivariate analysis

In multivariate analysis, only HIV disclosure, interpersonal communication patterns, and education variables were included in the modelling through bivariate selection (*p* < 0.25). Table 4 shows that for participants with higher education, the opportunity to have low-risk sexual behaviour was 2.807 times compared to participants with low education after controlling for the variables of HIV disclosure and interpersonal communication patterns. The multivariate test results in the last modelling revealed that the education variable had the highest OR value; therefore, it can be concluded that education was the dominant variable affecting the sexual risk behaviour of HIV-positive MSM in Batam.

## Discussion

### Correlation between HIV disclosure and sexual risk behaviour

In this study, low HIV disclosure was reported on 71 participants (56.3%) compared to the high HIV disclosure. This is in line with the study of Wei et al. in Asia, demonstrated that the prevalence of nondisclosure of HIV-positive MSM was higher, covering 67.3% of respondents [9]. The low level of HIV disclosure in HIV-positive MSM can be caused by a variety of factors, including fear of experiencing stigma, isolation, and misunderstanding or stress that can come from family, friends, and the environment after disclosure [23]. After disclosure, HIV-positive MSM had several positive and negative consequences [24]. Negative consequences can be in the form of social rejection or discrimination, including rejection from the family and stigma [24, 25]. Fear of stigma is considered inhibiting disclosure [25].

The results of bivariate analysis revealed a significant correlation between HIV disclosure and sexual risk behaviour (*p* = 0.019, α = 0.05). The results of this analysis are in line with the research of Wei et al.*,* where it was found that 86.5% of respondents who did not disclose their HIV status displayed sexual risk behaviour (67.5%) and had multiple sexual partners and UAI with these partners [9].

Condomless last anal intercourse (CLAI) after HIV serostatus disclosure was associated with a much lower risk of HIV exposure compared to nondisclosure, not using condoms, and not having a partner with effective antiretroviral therapy (ART) [11, 26]. In permanent partners, exposure to HIV through sexual risk intercourse can only be prevented by maintaining consistent condom use or by knowing the HIV status of each partner through HIV testing. Conversely, exposure to HIV with nonpermanent partners is mostly related to no disclosure of HIV status, so it can be prevented by oral chemoprophylaxis, and more frequent and consistent condom use and disclosure of serostatus HIV status [11].

### Correlation between interpersonal communication patterns and sexual risk behaviour

More than half participants (52.4%) had an open communication pattern with their partners. The results of statistical tests revealed a significant correlation between interpersonal communication patterns and sexual risk behaviour (*p* = 0.016, α = 0.05). This result is consistent with the research of Widman, Golin, and Noar in HIV-positive respondents, it found that sexual communication can predict a person’s sexual behaviour. Respondents with more sexual communication were less likely to have unprotected sex, both at the beginning and after a 6 month relationship [27]. The quality of good or excellent communication in relationships remains significantly related to sexual partners’ conversations about sexual behaviour regarding HIV prevention [28].

Qualitative research on MSM in New York City on how communication about HIV prevention before sex can result in safer sex practices and a reduction in HIV transmission to MSM find several key themes including; There is no opportunity to talk about HIV prevention, barriers to HIV prevention, concerns about contracting HIV, thinking about sexual health by asking the partner’s HIV status and using condoms [16]. The success of communication between individuals in determining a person’s decision to conduct positive behaviour is influenced by the characteristics of interpersonal communication, one of which is openness.

### The most associated variable to sexual risk behaviour

The final multivariate modelling test found that education was the dominant variable affecting the sexual risk behaviour of HIV-positive MSM. According to WFP (World Food Programme), increasing levels of education have a protective effect on HIV infection through changes to safer sexual behaviour. Education has different effects on different sexual behaviours, including condom use, having multiple sexual partners, and age of first sexual experience [29]. Educated individuals are more likely to act based on information about HIV prevention. When information about HIV prevention is provided, educated individuals change their intentions and become less likely to have multiple sexual partners [30].

The results of the multivariate analysis support the previous research on MSM in China, which showed that the main factor associated with HIV infection is the level of education. Respondents who were not highly educated were (49.39%) more likely to be diagnosed with HIV, which might be due to a low awareness of the risk of HIV and high-risk sexual behaviour [31]. The education variable in this study had a greater influence because at a higher level of education it could influence HIV positive MSM decisions in engaging in safe sexual behaviour. In this study, 43.6% HIV-positive MSM with high disclosure still displayed high-risk sexual behaviour. This might happen because younger HIV-positive MSM who are open about their HIV status with their sexual partners are more likely to be indicating UAI when opening up about their status with partners [12]. It can be concluded that education level has more influence on sexual risk behaviour because education is able to form a strong character in a person to maintain his desire to practice safe sex behaviour. Conversely, disclosure of HIV status in couples leads to inconsistent influence on sexual behaviour.

## Conclusion

There was a correlation between HIV disclosure and interpersonal communication patterns with sexual risk behaviour. Educational status was the most associated variable to sexual risk behaviour of HIV-positive MSM. Nurses as professional health workers are expected to improve nursing care for HIV-positive MSM, especially comprehensive assessment to obtain information about their risk behaviours in HIV transmission. Nurses need to improve patient personal counselling and plan specific learning model by involving HIV-positive MSM to reduce their risk behaviours. The results of this study form the basis for further research, and factors that can be further explored include the age of sexual initiation, partner drug use, and several others.

## Data Availability

Due to privacy data, all data and material is available if requested.

## Abbreviations

AIDS: Acquired Immune Deficiency Syndrome
UNAIDS: The Joint United Nations Programme on HIV/AIDS

## References

[1] UNAIDS (2017). UNAIDS Data.

[2] Dinas Kesehatan Kota Batam (2018). Data HIV/AIDS sampai dengan Juni 2018.

[3] Dinas Kesehatan Kota Batam (2018). Laporan konseling dan tes HIV sukarela (KTS/VCT) Tahun 2017.

[4] UNAIDS (2017). Global HIV statistics.

[5] Ministry of Health Republic of Indonesia (2016). HIV epidemiology review Indonesia.

[6] Rocha GM, Kerr LRFS, Kendall C, Guimarães MDC. Risk behavior score : a practical approach for assessing risk among men who have sex with men in Brazil. Braz J Infect Dis 2018; 2(2): 113–122. 10.1016/j.bjid.2018.02.008. 10.1016/j.bjid.2018.02.008 PMC9428223 29551334

[7] Grabovac I, Meilinger M, Schalk H, Leichsenring B, Dorner TE. Prevalence and associations of illicit drug and polydrug use in people living with HIV in Vienna. Sci Rep 2018; 8: 8046. 10.1038/s41598-018-26413-5. 10.1038/s41598-018-26413-5 PMC5966416 29795303

[8] Daskalopoulou M, Rodger A, Phillips AN, et al. Recreational drug use, polydrug use, and sexual behaviour in HIV-diagnosed men who have sex with men in the UK: results from the cross-sectional ASTRA study. Lancet HIV 2014; 1: e22–e31. 10.1016/S2352-3018(14)70001-3. 26423813

[9] Wei C, Lim SH, Guadamuz TE, Koe S. HIV disclosure and sexual transmission behaviours among an internet sample of HIV-positive men who have sex with men in Asia: implications for prevention with positives. AIDS Behav 2012; 16(7): 1970–1978. 10.1007/s10461-011-0105-x. 10.1007/s10461-011-0105-x PMC3340458 22198313

[10] Edwards-Jackson N, Phanuphak N, Van Tieu H (2012). HIV serostatus disclosure is not associated with safer sexual behaviour among HIV-positive men who have sex with men (MSM) and their partners at risk for infection in Bangkok, Thailand. AIDS Res Ther.

[11] Marcus U, Schink SB, Sherriff N, et al. HIV serostatus knowledge and serostatus disclosure with the most recent anal intercourse partner in a European MSM sample recruited in 13 cities: results from the Sialon-II study. BMC Infect Dis 2017; 17(730): 1–17. 10.1186/s12879-017-2814-x. 10.1186/s12879-017-2814-x PMC5702243 29178847

[12] Cook SH, Valera P, Wilson PA. HIV status disclosure, depressive symptoms, and sexual risk behaviour among HIV-positive young men who have sex with men. J Behav Med 2015; 38: 507–517. 10.1007/s10865-015-9624-7. 10.1007/s10865-015-9624-7 PMC4425621 25773478

[13] Fasoli F (2017). Gay straight communication. Oxford Research Encyclopedia of Communication.

[14] Liu Y, Wang J, Qian HZ, et al. Seeking male sexual partners via internet and traditional venues among chinese men who have sex with men: implications for HIV risk reduction interventions. AIDS Behav 2016; 20(10): 2222–2230. 10.1007/s10461-016-1371-4. 10.1007/s10461-016-1371-4 PMC7784627 27000143

[15] White Hughto JM, Pachankis JE, Eldahan AI, Keene DE.“You can’t just walk down the street and meet someone”: the intersection of social–sexual networking technology, stigma, and health among gay and bisexual men in the small city. Am J Mens Health 2017; 11(3): 726–736. 10.1177/1557988316679563. 10.1177/1557988316679563 PMC5393935 27885147

[16] Aholou TM, Drumhiller K, Nanin J, Sutton MY (2017). Opportunities for HIV prevention communication during sexual encounters with black men who have sex with men. AIDS Patient Care STDs.

[17] Rahim F, Thaha RM, Natsir S. Penyalahgunaan obat tramadol dan somadril terhadap perilaku seks beresiko komunitas gay Kota Makasar. J Promkes. 2014; repository.unhas.ac.id.

[18] Putra INAM (2018). Hubungan harga diri, HIV status *disclosure,* dan pengetahun tentang HIV terhadap perilaku seksual beresiko penularan HIV pada ODHA LSL di Kota Medan: Universitas Indonesia.

[19] Smith P, Schmidt S, Allensworth-Davies D, Saitz R (2010). A single-question screening test for drug use in primary care. Arch Intern Med.

[20] Olley BO, Ishola A (2016). A brief scale for HIV self disclosure: development, validity and reliability. IFE PsychologIA.

[21] Crenshaw AO, Christensen A, Baucom DH, Epstein NB, Baucom BRW (2017). Revised scoring and improved reliability for the communication patterns questionnaire. Psychol Assess.

[22] Fisher TD, Davis CM, Yarber WL, Davis SL (2010). Handbook of sexuality-related measures.

[23] Chen L, Lian D, Wang B. Factors associated with disclosing men who have sex with men (MSM) sexual behaviours and HIV-positive status: a study based on a social network analysis in Nanjing, China. PLoS One. 2018;13(4): 1–11. 10.1371/journal.pone.0196116. 10.1371/journal.pone.0196116 PMC5908074 29672596

[24] Lin X, Chi P, Zhang L, Zhang Y, Fang X, Qiau S, Li X. Disclosure of HIV serostatus and sexual orientation amomg HIV-positive men who have sex with men in China. Community Ment Health J. 2016;52:457–65. 10.1007/s10597-015-9879-z. 10.1007/s10597-015-9879-z PMC6234002 26002087

[25] Obermeyer CM, Baijal P, Pegurri E. Facilitating HIV disclosure across diverse settings: a review. Am J Public Health. 2011;101(6):1011–23. 10.2105/AJPH.2010.300102. 10.2105/AJPH.2010.300102 PMC3093267 21493947

[26] Santos-Hövener C, Zimmermann R, Kücherer C, Bätzing-Feigenbaum J (2014). Conversation about serostatus decreases risk of acquiring HIV: results from a case control study comparing MSM with recent HIV infection and HIV negative controls. BMC Public Health.

[27] Widman L, Golin C, Noar S. When do condom use intentions lead to actions? Examining the role of sexual communication on safer sexual behaviour among people living with HIV. J Health Psychol. 2013;18(4):507–17. 10.1177/1359105312446769. 10.1177/1359105312446769 PMC3612384 22689591

[28] Tobin KE, Yang C, Sun C, Spikes P, Latkin CA. Discrepancies between HIV prevention communication attitudes and actual conversations about HIV testing within social and sexual networks of African American men who have sex with men. Sex Transm Dis. 2014;41(4):221–6. 10.1097/OLQ.0000000000000112. 10.1097/OLQ.0000000000000112 PMC4516456 24622631

[29] World Food Programme. Literature review on the impact of education levels on HIV/AIDS prevalence rates, https://hivhealthclearinghouse.unesco.org/library/documents/literature-review-impact-education-levels-hivaids-prevalence-rates. [Accessed 18 June 2019].

[30] Zuilkowski S, Jukes MCH. The impact of education on sexual behaviour in sub-Saharan Africa: a review of the evidence. AIDS Care. 2012;24(5):562–76. 10.1080/09540121.2011.630351. 10.1080/09540121.2011.630351 22149322

[31] Ye M, Giri M (2018). Prevalence and correlates of HIV infection among men who have sex with men: a multi-provincial cross-sectional study in the southwest of China. HIV AIDS.

